# A transgenic mouse embryonic stem cell line for puromycin selection of V0_V_ interneurons from heterogenous induced cultures

**DOI:** 10.1186/s13287-022-02801-7

**Published:** 2022-03-28

**Authors:** Jennifer Pardieck, Manwal Harb, Shelly E. Sakiyama-Elbert

**Affiliations:** 1 Department of Biomedical Engineering, The University of Texas at Austin, 107 W Dean Keeton St., Austin, TX 78712-1139 USA; 2 Department of Biomedical Engineering, Washington University in St. Louis, St. Louis, MO USA

**Keywords:** Neuronal networks, V0_V_ spinal interneurons, Selectable transgenic mESCs

## Abstract

**Background:**

Spinal interneurons (INs) relay sensory and motor control information between the brain and body. When this relay circuitry is disrupted from injury or disease, it is devastating to patients due to the lack of native recovery in central nervous system (CNS) tissues. Obtaining a purified population of INs is necessary to better understand their role in normal function and as potential therapies in CNS. The ventral V0 (V0_V_) INs are excitatory neurons involved in locomotor circuits and are thus of interest for understanding normal and pathological spinal cord function. To achieve scalable amounts of V0_V_ INs, they can be derived from pluripotent sources, such as mouse embryonic stem cells (mESCs), but the resultant culture is heterogenous, obscuring the specific role of V0_V_ INs. This study generated a transgenic mESC line to enrich V0_V_ INs from induced cultures to allow for a scalable, enriched population for future in vitro and in vivo studies.

**Methods:**

The transgenic Evx1-PAC mESC line was created by CRISPR-Cas9-mediated insertion of puromycin-*N*-acetyltransferase (PAC) into the locus of V0_V_ IN marker *Evx1*. *Evx1* and *PAC* mRNA expression were measured by qPCR. Viability staining helped establish the selection protocol for V0_V_ INs derived from Evx1-PAC mESCs inductions. Immunostaining was used to examine composition of selected inductions. Cultures were maintained up to 30 days to examine maturation by expression of mature/synaptic markers, determined by immunostaining, and functional activity in co-cultures with selected motor neurons (MNs) and V2a INs on microelectrode arrays (MEAs).

**Results:**

V0_V_ IN inductions were best selected with 4 µg/mL puromycin on day 10 to 11 and showed reduction of other IN populations and elimination of proliferative cells. Long-term selected cultures were highly neuronal, expressing neuronal nuclear marker NeuN, dendritic marker MAP2, pre-synaptic marker Bassoon, and glutamatergic marker VGLUT2, with some cholinergic VAChT-expressing cells. Functional studies on MEAs showed that co-cultures with MNs or MNs plus V2a INs created neuronal networks with synchronized bursting.

**Conclusions:**

Evx1-PAC mESCs can be used to purify V0_V_ IN cultures for largely glutamatergic neurons that can be used in network formation studies or for rodent models requiring transplanted V0_V_ INs.

**Supplementary Information:**

The online version contains supplementary material available at 10.1186/s13287-022-02801-7.

## Background

Spinal cord injury (SCI) persists as a devastating infliction for patients as doctors and researchers have yet to find a widely efficacious method to treat injured spinal cord tissue. An underlying hinderance in SCI treatment is the loss of neurons, which form connections between the body and the brain, as they are post-mitotic and have limited regenerative capacity. To reconnect the lost circuits, cell transplantation is a potential method of introducing cells in and around the injury site to support growth of spared neurons through release of neurotrophic factors and for cell replacement. For cell transplantation, several cell lineages have been investigated to determine their contribution to repair (see this review for more information: [1]), but the propriospinal, interneuron (IN) populations have been shown to create local connections post-injury [2–4]. INs are classified as dorsal or ventral, which contribute largely to sensory or motor function, respectively [5], and several studies have worked toward deconstructing the role of the different IN subtypes and the interplay between them, which drives normal function [6], as well as their potential as therapeutic populations [1]. Many of these studies utilized knockdown or knockout animal models, but such methods may obscure the functional contribution of a population through possible redundancies or compensations inherent to spinal circuits [7, 8], requiring additional, alternative methods using particular IN populations in isolation. Such isolation would allow for more controlled studies but would require some method of purification for the population of interest from its source.

V0 INs are of interest as a therapeutic population. They comprise a diverse set of INs that arise from a Dbx1^+^ progenitor pool [9, 10], and include excitatory, ventral V0 (V0_V_) INs, which express transcription factor Evx1 [11], and inhibitory, dorsal V0 (V0_D_) INs, with both ipsilateral and commissural projecting axons. V0 INs contribute to left–right alternation [12, 13], with V0_D_ INs shown to contribute more at lower locomotor frequencies and V0_V_ INs contribute at higher frequencies in rodents [13]. A subset of V0_V_ INs expresses the transcription factor Pitx2 and includes an ipsilateral population that project onto motor neurons (MNs) [14]. These Pitx2^+^ cells include the cholinergic V0_C_ and glutamatergic V0_G_ populations, with V0_C_ involved in task-dependent excitation of MNs [14]. The contribution of V0_V_ INs as an excitatory population involved in locomotor circuits, including cells that project onto MNs, is therefore of great appeal for cell replacement therapies.

For an abundant source of INs, one could differentiate them from pluripotent stem cells (PSCs), including patient-derived induced PSCs (iPSCs) and embryonic stem cells (ESCs), into the desired neuronal type. Induction protocols to obtain neurons from PSCs, however, are not completely efficient—often with less than half the culture comprising the cell type of interest—thus requiring a method to purify the intended population from the heterogenous culture. For example, our recent induction protocol developed to generate V0_V_ INs from mouse ESCs (mESCs) yields approximately 40% of the cells expressing V0_V_ IN markers 2 days post-induction [15]. To purify the cell population of interest from such heterogenous inductions, our laboratory has generated transgenic mESC lines that use an IN- or MN-specific gene marker to drive expression of a puromycin resistance enzyme, puromycin-N-acetyltransferase (PAC) [16–19]. As the induced mESCs become the intended INs expressing their specific gene marker, it drives expression of PAC and thus renders those cells insensitive to puromycin exposure, while other cells in the culture do not survive. In this article, a mESC line used to select V0_V_ INs by insertion of PAC into the Evx1 locus is analyzed for efficiency of selection, remaining proliferative cells and other IN populations. Maturation and electrophysiological characterization of selected V0_V_ IN populations are also assessed.

## Materials and methods

### Cell culture media formulations

*Complete medium (CM)* Dulbecco’s Modified Eagle Medium (DMEM; Thermo Fisher Scientific, 11965; +l-Glutamine, hi glucose) containing 1x nucleoside solution (10 μM thymidine, and 30 μM of adenosine, cytosine, guanosine, and uridine), 10% fetal bovine serum, and 10% newborn calf serum.

*DFK5 medium* DMEM/F12 (Thermo Fisher Scientific, 11320; +l-Glutamine, +Sodium Pyruvate, −HEPES, hi glucose) containing 0.5x nucleoside solution (5 μM thymidine, 15 μM of adenosine, cytosine, guanosine, and uridine), 5% knockout serum replacement (Thermo Fisher Scientific, 10828), 0.5x non-essential amino acid solution (50 µM of each amino acid), 1x Insulin, Transferrin, Selenium Solution (Thermo Fisher Scientific, 41400; 1.72 µM Insulin, 68.8 nM Transferrin, 38.7 nM Sodium Selenite), and 55 µM BME.

*Neuronal medium* DFK5 medium and Neurobasal medium (NB; Thermo Fisher Scientific, 21103049) mixed in a 1:1 *v*/*v* ratio.

*Long-term medium* NB with 1x GlutaMAX (Thermo Fisher Scientific, 35050061), 1x B-27 supplement (Thermo Fisher Scientific, 17504044), and 5 ng/mL for each of brain-derived neurotrophic factor (BDNF, recombinant human, PeproTech, 450-02: resuspended as a 10 µg/mL stock in 0.1% bovine serum albumin [BSA; MilliporeSigma, A2058] in phosphate buffered saline [PBS]), glial cell line-derived neurotrophic factor (GDNF, recombinant human, PeproTech, 450-10: resuspended as a 10 µg/mL stock in 0.1% BSA in PBS), and neurotrophin-3 (NT-3, recombinant human, PeproTech, 450-03: resuspended as a 10 µg/mL stock in 0.1% BSA in PBS). 0.1% BSA was prepared by diluting powder in PBS and then filtering using Steriflip-GP Sterile Centrifuge Tube Top Filter Unit (MilliporeSigma, SCGP00525).

### ESC maintenance

RW4 mESCs (ATCC, SCRC-1018), Hb9-puro, Chx10-puro-tdTomato and Evx1-PAC mESCs were maintained in T-25 flasks coated with 0.1% gelatin (MilliporeSigma, G1393; in water) in CM containing 1000 U/mL leukemia inhibitory factor (LIF; MilliporeSigma, ESG1106) and 100 μM β-mercaptoethanol (BME; Thermo Fisher Scientific, 21985023) in 5% CO_2_ at 37 °C. For passaging, mESC colonies were dissociated with 0.25% trypsin ethylenediaminetetraacetic acid (trypsin–EDTA; Thermo Fisher Scientific, 25200072) for 5 min, followed by quenching and trituration with CM. Single cells were plated in a new flask containing CM +LIF +BME at a 1:5 ratio and grown for two days or until ~ 80% confluent.

### Design and generation of vectors used to create *Evx1*-PAC selectable mESC line

#### Evx1 guide RNA vectors

A list of potential guide RNA sequences to the *Evx1* locus was generated by providing a partial *Evx1* genomic DNA input sequence to the algorithm found on the CRISPOR website [20] (results can be found here: http://crispor.tefor.net/crispor.py?batchId=mN1FkRligAThyqKv3eg3). Two guide RNA oligonucleotide sequences were selected based on their proximity to the *Evx1* transcript start codon as well as low number of off-target sequences listed in CRISPOR results.

Guide RNA oligonucleotides were designed and generated according to the Joung lab gRNA cloning protocol provided on the Addgene website [21] by annealing phosphorylated single-stranded oligonucleotide sequences (ordered from Integrated DNA Technologies; see Table 1). Briefly, equivalent molar amounts of top and bottom oligonucleotide sequence strands were mixed in T4 DNA Ligase buffer (New England Biolabs [NEB], B0202S) including T4 Polynucleotide Kinase enzyme (NEB, M0201S). Oligonucleotide mixtures were incubated at 37 °C for 1 h to enable phosphorylation, followed by heating to 95 °C for 5 min and cooling at a rate of − 5 °C/min to 10 °C to allow annealing.

**Table 1:** Oligonucleotides and primer sequences

Oligonucleotide name	Sequence
Evx1 guide 21 F	ACACCTCGCGGGCTCAGGCAACCGTG
Evx1 guide 21 R	AAAACACGGTTGCCTGAGCCCGCGAG
Evx1 guide 28 F	ACACCAAGTCGGAGGGCCGGCTCGCG
Evx1 guide 28 R	AAAACGCGAGCCGGCCCTCCGACTTG
5′ Evx1 homology arm F	GGGGACAAGTTTGTACAAAAAAGCAGGCTCTGTAGCTATGGATTCT
5′ Evx1 homology arm R	CTCGGTCATATTGGCAAATTAGAGACCCTC
PAC cassette F	GAGGGTCTCTAATTTGCCAATATGACCGAG
PAC cassette R	GTCTGAGCTACTAGTCTAGAACTAGTGGATCC
3′ Evx1 homology arm F	GGATCCACTAGTTCTAGACTAGTAGCTCAGAC
3′ Evx1 homology arm R	GGGGACCACTTTGTACAAGAAAGCTGGGTTCGGTCCTTTATATTC
5′ genomic Evx1 F	GGAACGGGTACTTTAGGCTC
5′ PAC insertion R	TCGTAGAAGGGGAGGTTGC
3′ PAC/Neo insertion F	TCTGGATTCATCGACTGTGG
3′ genomic Evx1 R	TTGGGACCATTTCCGACCTG

MLM3636 vector (Addgene, 43860) was cut with BsmBI restriction enzyme (NEB, R0580S) in 1x NEBuffer 3.1 (NEB, B7203S) for 1 h at 55 °C and then deactivated at 80 °C for 20 min. Digested MLM3636 vector was dephosphorylated with shrimp alkaline phosphatase (rSAP; NEB, M0371S) in 1x CutSmart Buffer (NEB, B7204S) at 37 °C for 30 min, followed by deactivation of rSAP at 65 °C for 10 min.

Vectors for *Evx1* guide 21 and *Evx1* guide 28 (see Table 1) were created by ligating the annealed oligonucleotide sequences with the digested MLM3636 vector using T4 DNA ligase for 16 h at 16 °C. Ligations were transformed in DH5α *E. coli* and selected on 2% agar in Lennox LB broth (MilliporeSigma, L3022) containing 50 µg/mL ampicillin (MilliporeSigma, A0166: resuspended as a 50 mg/mL stock in water).

#### *Evx1*-PAC donor vector

PCR with Klentaq LA (DNA Polymerase Technology, 110) was used to amplify both the *PAC*-PGK-*Neo* cassette from existing vectors used to create other selectable lines in our laboratory [18, 19] as well as genomic DNA sequences flanking the *Evx1* guide RNA sequences for 5′ and 3′ *Evx1* homology arms using genomic DNA template from the RW4 mESC line (see Table 1 for primers). The homology arm primers included attB sites to allow for Gateway recombination cloning. Overlap extension PCR was used to amplify the 5′ homology arm with the PAC cassette and then the 3′ homology arm. BP clonase (Thermo Fisher Scientific, 11789020) was used to recombine the homology arm-flanked PAC overlap extension PCR product into the pDONR221 vector (Thermo Fisher Scientific, 12536017). DNA from bacterial clones selected on LB-agar plates containing 50 µg/mL Kanamycin (Thermo Fisher Scientific, BP906: resuspended as a 50 mg/mL stock in water) were isolated using QIAprep Spin Miniprep Kit (Qiagen, 27106) and sequence-verified (Sanger sequencing at UT Austin core facility), then recombined into the pWS-TK3 vector (Addgene, 20349) using LR clonase (Thermo Fisher Scientific, 11791020) according to manufacturer instructions. Bacterial clones containing the final pWS-TK3-Evx1-PAC donor vector were selected on LB-agar plates containing ampicillin and isolated using QIAprep Spin Miniprep Kit.

### Electroporation and expansion of *Evx1*-PAC mESC clones

1 × 10^7^ RW4 mouse embryonic stem cells were dissociated with 0.25% trypsin–EDTA, quenched with CM, and pelleted at 300 × *g*. Two plasmid mixtures were made, one for each of the guide RNA vectors: 10 µg of pWS-TK3-Evx1-PAC, 1 µg of SpCas9 vector (Addgene, 43945), and 1 µg of either of the MLM3636-guide RNA vectors (guide 21 or 28) were mixed with 800 µL electroporation buffer (20 mM HEPES pH 7.5, 137 mM NaCl, 5 mM KCl, 0.7 mM Na2HPO4, 6 mM dextrose) and used to resuspend the RW4 mESCs. Each cell mixture was loaded into a chilled 0.4 cm electroporation cuvette (Bio-Rad, 1652081), mounted into the Gene Pulser Xcell Eukaryotic System (Bio-Rad, 1652661), and electroporated with voltage parameter 0.23 kV and capacitance parameter 960 µF. After pulsing, cells were quickly moved into CM +LIF +BME and grown in a 0.1% gelatin-coated 10 cm non-tissue culture treated dish for 2 days.

From days 2 to 10, electroporated cells were grown in CM +LIF +BME with 40 µg/mL Geneticin/G418 (Thermo Fisher Scientific, 10131027) for positive selection and 150 nM fialuridine (MilliporeSigma, SML0632) for negative selection; medium was replaced every 2 days. At day 10, visible single colonies were picked and dissociated using trypsin–EDTA into 96 well 0.1% gelatin-coated cell culture plates, quenched with CM +LIF +BME, and once confluent, dissociated into two wells of a 96 well plate; one well was used to genotype clones for insertion of *PAC* into the *Evx1* genomic locus using junction PCR; see Table 1 for primer sequences). Clones positive for PAC by jPCR were also tested by copy number assay using qPCR with Tert (Thermo Fisher Scientific, 4458368) as the reference control, GAPDH (Thermo Fisher Scientific, Mm00186825_cn) as the reference for 2 copies in RW4 mESCs, and a PAC custom assay (forward sequence: GGTGCCCGCCTTCCT; reverse sequence: CGGCGGTGACGGTGAA) using Hb9-puro mESCs as a reference for 1 copy and analyzed using the CopyCaller v2.1 software. Clones with single copies of *PAC* were expanded in different well sizes until 80% confluent in T75 flasks (Thermo Fisher Scientific, 07202000), and then, cells were dissociated in trypsin–EDTA, quenched with CM, pelleted at 300 × *g*, resuspended in cell freezing medium (MilliporeSigma, C6295), and frozen in a Mr. Frosty freezing container (Thermo Fisher Scientific, 51000001) at − 80 °C before storage in liquid nitrogen.

### Cre excision of PGK-*Neo*

In one Evx1-PAC mESC clone verified by junction PCR (jPCR) and copy number assay, the PGK-*Neo* selection cassette flanked by loxP sites was excised by transfecting the mESCs with pTurbo-Cre (a gift from Dr. Timothy Ley) using Lipofectamine 3000 (Thermo Fisher Scientific, L3000001). The day before transfection, Evx1-PAC mESCs were dissociated using trypsin–EDTA, quenched with CM, and then 5 × 10^5^ cells were plated in a 6-well plate well and grown in CM +LIF +BME. For transfection, 3.75 µL of Lipofectamine 3000, 10 µL of P3000 Reagent, 5 µg of pTurbo-Cre plasmid were mixed in Opti-MEM medium (Thermo Fisher Scientific, 31985062) and added to the mESC culture. Once colonies were visible, clones were picked and dissociated into 96-well plates. Each was evenly split among two wells to test for sensitivity to G418 added to one of the two wells to observe cell death. Those that showed cell death were chosen for jPCR to look for the presence of *Neo* in the *Evx1* locus. Two clones that no longer contained the PGK-*Neo* cassette were chosen, expanded, frozen down, and one was used for all further studies.

### V0_V_ IN induction, selection, and long-term culture

For V0_V_ IN induction, mESCs were cultured as previously described [15]. Briefly, mESCs were dissociated, pelleted, and resuspended in DFK5 medium in a 10 cm tissue culture-treated agar-coated dish for embryoid body (EB) formation. After 2 days (2^−^), EBs were settled and fresh DFK5 medium was used to return EBs to the agar-coated dish. After another 2 days (4^−^), EBs (2.5 mL per 10 cm plate) were settled and resuspended in 10 mL of fresh DFK5 + 1 µM all-*trans* retinoic acid (RA; MilliporeSigma, R2625: resuspended as a 20 mM stock in dimethyl sulfoxide [DMSO; MilliporeSigma, D2650]) + 100 nM purmorphamine (purm; MilliporeSigma, 540223). After 2 days (4^−^/2^+^), medium was replaced with 10 mL of fresh DFK5 + 1 µM RA + 5 µM *N*-{*N*-(3,5-difluorophenacetyl-l-alanyl)}-(*S*)-phenylglycine-t-butyl-ester (DAPT; MilliporeSigma, D5942: resuspended as a 10 mM stock in DMSO). Induction was complete after another two days (4^−^/4^+^), at which time cultures were dissociated with 0.25% trypsin–EDTA, quenched with CM, passed through a 100 µm cell strainer (Corning, 352360), counted, and plated on laminin-coated wells at 1 × 10^5^ cells/cm^2^ in neuronal medium supplemented with 1x GlutaMAX, 1x B-27 supplement, and 5 ng/mL for each of BDNF, GDNF, and NT-3.

For selection conditions, cultures were exposed to 4 µg/mL puromycin dihydrochloride (puro; MilliporeSigma, P8833: resuspended as a 10 mg/mL stock in water) in supplemented neuronal medium. After 24 h, medium was aspirated, cells were rinsed with NB and cultured in neuronal medium with supplements replaced every two days. For longer-term studies, after day 12, cultures were fed with half-volume exchanges of long-term medium every two days until their respective end points.

### V2a IN and MN induction and selection

Chx10-puro-tdTomato mESCs were induced using a 2^−^/4^+^ protocol using 10 nM RA and 1 μM purm from days 2 to 6 and 5 μM DAPT for days 4 to 6 [22]. On day 6, V2a IN EBs were settled, induction medium aspirated, and neuronal medium with supplements was used to move EBs back to the 10 cm dish. 2 µg/mL puro was added to the culture for 24 h. Hb9-puro mESCs were induced using the MN 2^−^/4^+^ induction protocol using 2 μM RA and 0.5 μM SAG from days 2 to 6 [23]. Day 6 EBs were cultured in neuronal medium with supplements containing 4 µg/mL puro from day 6 to 7.

### Microelectrode array co-cultures of selected V0_V_ INs with selected mESC-derived V2a IN and MNs

CytoView 24-well microelectrode array (MEA) plates (Axion Biosystems, M384-tMEA-24W-5) were used to culture 1 × 10^5^ cells/array of V0_V_ INs, V2a INs, MNs, or a combination of V0_V_ INs with V2a INs or MNs, V2a INs and MNs, or all three populations together. Array areas were coated with 0.01% poly-l-ornithine (MilliporeSigma, P3655; in 10 mM borate buffer, pH 8.3), followed by washing 2 times with excess HEPES-buffered saline solution (HBSS, pH 7.2) and coating with 5 µg/mL laminin (mouse; Thermo Fisher Scientific, 23017015; in HBSS). To measure activity, CytoView plates were loaded into a Maestro Edge instrument (Axion Biosystems) for recordings using AxIS Navigator software.

V0_V_ IN induction cultures were selected on day 10 with 4 µg/mL puro in supplemented neuronal medium for 24 h. On day 11, selection medium was aspirated, wells were washed with NB, and medium was replaced with supplemented neuronal medium. On day 12 of V0_V_ IN culture, V0_V_ INs were lifted with Accutase solution (MilliporeSigma, A6964), quenched with CM, spun at 400 × *g* and resuspended in 1 mL neuronal medium with supplements. Day 6–7 selected cultures of V2a INs and/or MNs were dissociated with 0.25% trypsin–EDTA, quenched with CM, strained through 100 µm cell strainers, spun at 400 × *g*, and resuspended in 1 mL neuronal medium with supplements. All populations were then counted to achieve cell densities of 1 × 10^5^ cells/10 µL drop to load onto MEA wells, which have a 22.9 mm^2^ surface area; for single-population MEA cultures, cells were plated at 1 × 10^5^ cells/array, for double-population cultures, each population was plated at 5 × 10^4^ cells/array, and for triple-population cultures, each population was plated at 3.3 × 10^4^ cells/array. After allowing attachment for 4 h, 300 µL of long-term medium was added slowly to each well; medium was replaced by half-volumes every 2 days.

To detect spontaneous firing of action potentials in MEA cultures, AxIS Navigator software parameters were set in the spike detector. Peak detection with adaptive threshold was used with a set threshold of 5× standard deviation to detect only negative inflections of spikes that crossed this threshold. Durations for pre- and post-spike detection were set to 1 ms and 2 ms, respectively, and the spike detection interval was set to 1.6 ms to enable collection of 20,000 samples for each spike.

### Isolation of RNA, reverse transcription, and qPCR

To collect cultured cells for qPCR analysis, medium was aspirated and cells were detached by addition of 0.25% trypsin–EDTA, followed by quenching and dissociation in CM. Cells were pelleted at 300 × *g*. Medium was aspirated, and pellets were resuspended in RLT buffer as provided from the RNeasy Mini Kit (Qiagen, 74106). Pellets were either frozen at − 80 °C or immediately used with the RNeasy kit using on-column DNase (Qiagen, 79254) to isolate RNA per manufacturer instructions. 500 ng of RNA was reverse transcribed using the High-Capacity cDNA Reverse Transcription Kit (Thermo Fisher Scientific, 4368813) per manufacturer instructions.

For qPCR, a solution of ultrapure water, TaqMan Fast Advanced Master Mix (Thermo Fisher Scientific, 4444963), a TaqMan probe against mouse *β-actin* as a reference gene (Thermo Fisher Scientific, Mm02619580_g1, using VIC-MGB_PL dye), and the TaqMan probe against the target gene using FAM-MGB dye (*PAC* [custom ordered]: forward sequence: GGTGCCCGCCTTCCT; reverse sequence: CGGCGGTGACGGTGAA; *Evx1*: Mm00433154_m1) was prepared and loaded into a MicroAmp Fast Optical 96-Well Reaction Plate (Thermo Fisher Scientific, 4346906) before loading each sample in triplicate. Plates were sealed, spun briefly to remove bubbles, and loaded into the QuantStudio 3 instrument for measurement. The fold changes in mRNA expression levels were calculated using 2^^−ΔΔCT^ values with *β-actin* as the reference gene relative to mESCs.

### LIVE/DEAD staining to determine selection window by image analysis

Cultures were grown in 48-well plates for image analysis. To test selection, 4 µg/mL puro was added on days 9, 10, or 11 for 24 h, followed by a rinse with NB before culture in neuronal medium with supplements for an additional 2 days post-selection before staining and imaging.

The LIVE/DEAD Viability/Cytotoxicity Kit for mammalian cells (Thermo Fisher Scientific, L3224) was used with Calcein AM at 0.4 µM (live stain, 1:10,000 dilution in PBS) and Ethidium homodimer-1 at 1 µM (dead stain, 1:2000 dilution in PBS) to stain cultures that included unselected cells and cells selected at different time points. Cells were incubated for 30–40 min before rinsing once with PBS, then imaging.

Images were analyzed using a CellProfiler pipeline [24, 25] to determine the percentage of living cells. Puro results in early termination of protein translation to lead to cell death, resulting in cell debris that is removed during media changes, therefore the majority of dead cells were washed away post-selection. 4 images were taken of each well for each condition at each time point with *N* = 6. The percentage of living cells was calculated based on the count of live cells in selected wells divided by the count of live cells in unselected wells, as all wells were plated at the same density of 1 × 10^5^ cells/cm^2^.

### Flow cytometry

Day 11 cultures, both unselected and selected with 4 µg/mL puro from day 10–11, were dissociated and spun at 400 × *g* for 5 min, and pellets were fixed in 4% paraformaldehyde (PFA; MilliporeSigma, P6148; in 0.1 M phosphate buffer) for 20 min at room temperature. Cells were then incubated in 2% normal goat serum (NGS; MilliporeSigma, G9023) with 0.1% Triton X-100 (MilliporeSigma, X100) in PBS to permeabilize and block for 30 min at room temperature. Primary antibodies (see Table 2) were diluted in 1% NGS in PBS and cells were stained for 40 min at room temperature. Cells were washed 2 times for 10 min per wash with PBS. Secondary antibodies (see Table 3) were diluted in 1% NGS in PBS and cells were stained for 40 min at room temperature. Cells were washed 2 times for 10 min per wash and then resuspended in PBS before running samples on Attune NxT cytometer (Thermo Fisher Scientific).

**Table 2:** Primary antibodies used for ICC and flow cytometry

Target	Final concentration or dilution used	Source, product number
βIII tubulin	1 µg/mL	Biolegend, 802001
βIII tubulin	1 µg/mL	Biolegend, 845501
Chx10	0.2 µg/mL	Santa Cruz Biotechnology, Inc., sc-374151
Dmrt3	2 µg/mL	Thermo Fisher Scientific, BS-4264R
En1	2 µg/mL	Developmental Studies Hybridoma Bank, 4G11
Evx1	0.5 µg/mL	Developmental Studies Hybridoma Bank, 99.1-3A2^a^
Hb9	2 µg/mL	Developmental Studies Hybridoma Bank, 81.5C10
Lim1	5 µg/mL	Developmental Studies Hybridoma Bank, 4F2
MAP2	1:1000 dilution	MilliporeSigma, AB5622
NeuN	1:50 dilution	MilliporeSigma, MAB377
Olig2	1:500 dilution	MilliporeSigma, MABN50
VAChT	5 µg/mL	Thermo Fisher Scientific, MA5-27662
VGLUT2	1:3000 dilution	MilliporeSigma, AB2251-I

**Table 3:** Secondary antibodies used for ICC and flow cytometry

Target^a^	Conjugated fluorophore	Final concentration^b^ (µg/mL)	Product number^c^
Guinea pig IgG	Alexa Fluor 488	4	A-11073
Mouse IgG1	Alexa Fluor 488	2, 4	A-21121
Mouse IgG1	Alexa Fluor 647	4	A-21240
Mouse IgG2a	Alexa Fluor 488	2, 4	A-21131
Mouse IgG2a	Alexa Fluor 555	4	A-21137
Mouse IgG2a	Alexa Fluor 647	2, 4	A-21241
Mouse IgG2b	Alexa Fluor 488	2	A-21141
Rabbit IgG	Alexa Fluor 488	2, 4	A-11008
Rabbit IgG	Alexa Fluor 647	2, 4	A-21244

^a^All secondary antibodies were from goat hosts

^b^2 µg/mL was used for flow cytometry, 4 µg/mL was used for ICC

^c^All secondary antibodies were purchased from Thermo Fisher Scientific

### Immunocytochemistry and image analysis

Long-term cultures in 48-well, laminin-coated plates were used for immunocytochemistry (ICC) analysis. After aspirating culture medium, wells were rinsed with PBS, and cells were fixed in 4% PFA for 20 min at room temperature. Cells were then incubated in 2% normal goat serum (NGS; MilliporeSigma, G9023) with 0.1% Triton X-100 (MilliporeSigma, X100) in PBS to permeabilize and block for 30 min at room temperature. Primary antibodies (see Table 2) were diluted in 1% NGS in PBS and cells were stained overnight at 4 °C. Wells were washed 3 times for 10 min per wash with PBS. Secondary antibodies (see Table 3) were diluted in 1% NGS in PBS and filtered through a 0.22 µm PVDF syringe filter (MilliporeSigma, SLGV033RS). To prevent fluorescence photobleaching of conjugated secondary antibodies, plates were wrapped in foil, while cells were incubated for 1 h at room temperature. Secondary antibody solutions were removed, and cells were washed 3 times for 10 min per wash with PBS. Cells were then stained in 1:1000 Hoechst 33258 (Thermo Fisher Scientific, H3569) in PBS for 10 min, washed once with PBS, then imaged on a DMi8 inverted widefield microscope (Leica).

### Statistics

GraphPad Prism version 7 and Microsoft Excel were used for statistical analyses. In Prism, ROUT method with *Q* = 1% was used to remove outliers—this method initially generates a Lorenzian distribution and uses a robust curve fit and Q as a false discovery rate to determine outliers from the residuals of the fit, and then for the remaining data, it uses least-squares regression to determine any additional outliers. Values are reported as means and error bars are standard error of the mean (S.E.M.) unless otherwise stated. One-way analysis of variance (ANOVA) using Scheffe’s multiple comparison method with 95% confidence was used to determine significance, which is indicated in figures as follows: * for *p* < 0.05, ** for *p* < 0.01, *** for *p* < 0.001, and **** for *p* < 0.0001, unless otherwise stated.

## Results

### Inserting PAC into a single *Evx1* gene locus

**Fig. 1:**
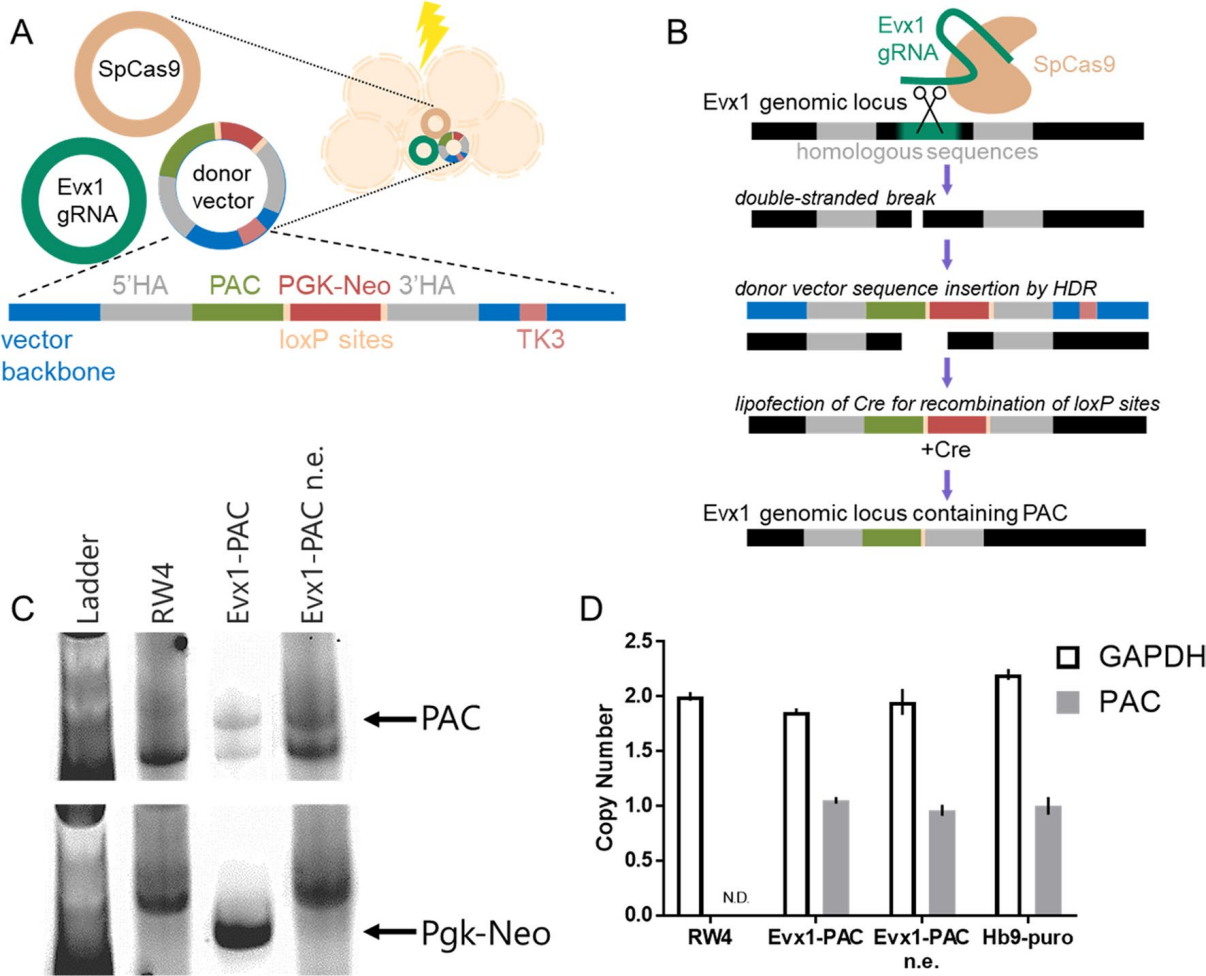
Inserting *PAC* into a single *Evx1* locus using CRISPR/Cas9-mediated homology-directed repair. **A** Schematic showing electroporation of mESCs to incorporate 3 plasmids: SpCas9, *Evx1* guide RNA (gRNA), and the pWS-TK3-Evx1-PAC donor vector. The donor vector contains homology arms (5′ and 3′ HAs) against *Evx1* genomic sequences flanking the cut site, *PAC*, floxed PGK-*Neo* for positive selection, and the negative selection marker TK3. **B** Schematic showing SpCas9 being guided to the *Evx1* genomic locus by *Evx1* gRNA. The Cas9 cut site is flanked by homologous sequences to the HAs of the donor vector, which is inserted into the double-stranded break (DSB) through homology-directed repair (HDR). Cre-mediated recombination removes the *Neo* cassette so that only *PAC* remains in the *Evx1* locus. **C** jPCR images showing the wildtype RW4 mESCs as a control, with PAC insertion in both Evx1-PAC and *Neo*-excised (n.e.) Evx1-PAC, as well as presence of *Neo* in Evx1-PAC and absence in Evx1-PAC n.e. mESCs. **D** Copy number assay using the previously established Hb9-puro mESC line as a control for one copy of *PAC* and RW4 mESCs as a control for two copies of *GAPDH* as a reference. Evx1-PAC and Evx1-PAC n.e. mESCs show one copy of *PAC*. Error bars show the range of possible copies, not S.E.M

V0_V_ IN marker-expressing cells and other propriospinal IN populations can be induced from mESCs, but the resulting culture is heterogenous and can include proliferative cell types that will dilute the post-mitotic neurons. To better characterize the derived V0_V_ IN population for use in controlled in vitro studies and potentially as a transplantable population in rodent SCI models, a method of purification is desirable. To this end, a gene for an enzyme that confers protection against puromycin (puro), PAC, was inserted into the locus of *Evx1*, the distinguishing marker for V0_V_ INs. The goal was to use CRISPR/Cas9-mediated homology-directed repair (HDR) to insert the *PAC* selection marker gene into a single locus to confer puro resistance while maintaining *Evx1* expression to enable V0_V_ IN induction (Fig. 1).

Three plasmids were electroporated into wildtype RW4 mESCs to promote HDR: one with a guide RNA (gRNA) to direct Cas9 to an appropriate site in the *Evx1* locus to create a double-stranded break (DSB), one encoding the SpCas9 enzyme, and a vector to act as donor DNA to insert *PAC* into the locus during HDR (Fig. 1A). The donor vector included homology arms—with sequences homologous to the genomic locus of *Evx1* adjacent to the guide RNA sequences in the genome—flanking *PAC* and floxed PGK-*Neo*, a positive selection marker; the vector backbone also included a negative selection marker, thymidine kinase 3 (*TK3*) to select against random, non-HDR-mediated insertion. After Cas9 generated a DSB at the site specified by the *Evx1* gRNA, the donor vector provided the donor DNA for DNA repair mechanisms to perform HDR (Fig. 1B). Junction PCR (jPCR) using a primer aligned to *Evx1* genomic sequence upstream of the insertion and a primer aligned to the *PAC* sequence was used to confirm insertion into the *Evx1* locus (Fig. 1C). To ensure that the primary transcript being expressed was *PAC* in the generated Evx1-PAC mESCs, the floxed PGK-*Neo* sequence was excised through Cre-mediated recombination; Cre was introduced by transfecting positive Evx1-PAC mESC clones screened by jPCR and determining their sensitivity to G418 (Neomycin). G418-sensitive clones were screened by jPCR using a primer aligned to *Neo* and a primer aligned to *Evx1* genomic sequence downstream of the cassette insertion to confirm removal of PGK-*Neo* (Fig. 1C). Using screened clones of Evx1-PAC and *Neo*-excised Evx1-PAC (Evx1-PAC n.e.) mESCs from jPCR, a copy number assay was performed to ensure that only one *Evx1* locus was modified (Fig. 1D). A single verified clone was used in all further experiments and is hereafter referenced as Evx1-PAC in all data and discussion.

### Determining puromycin selection window

**Fig. 2:**
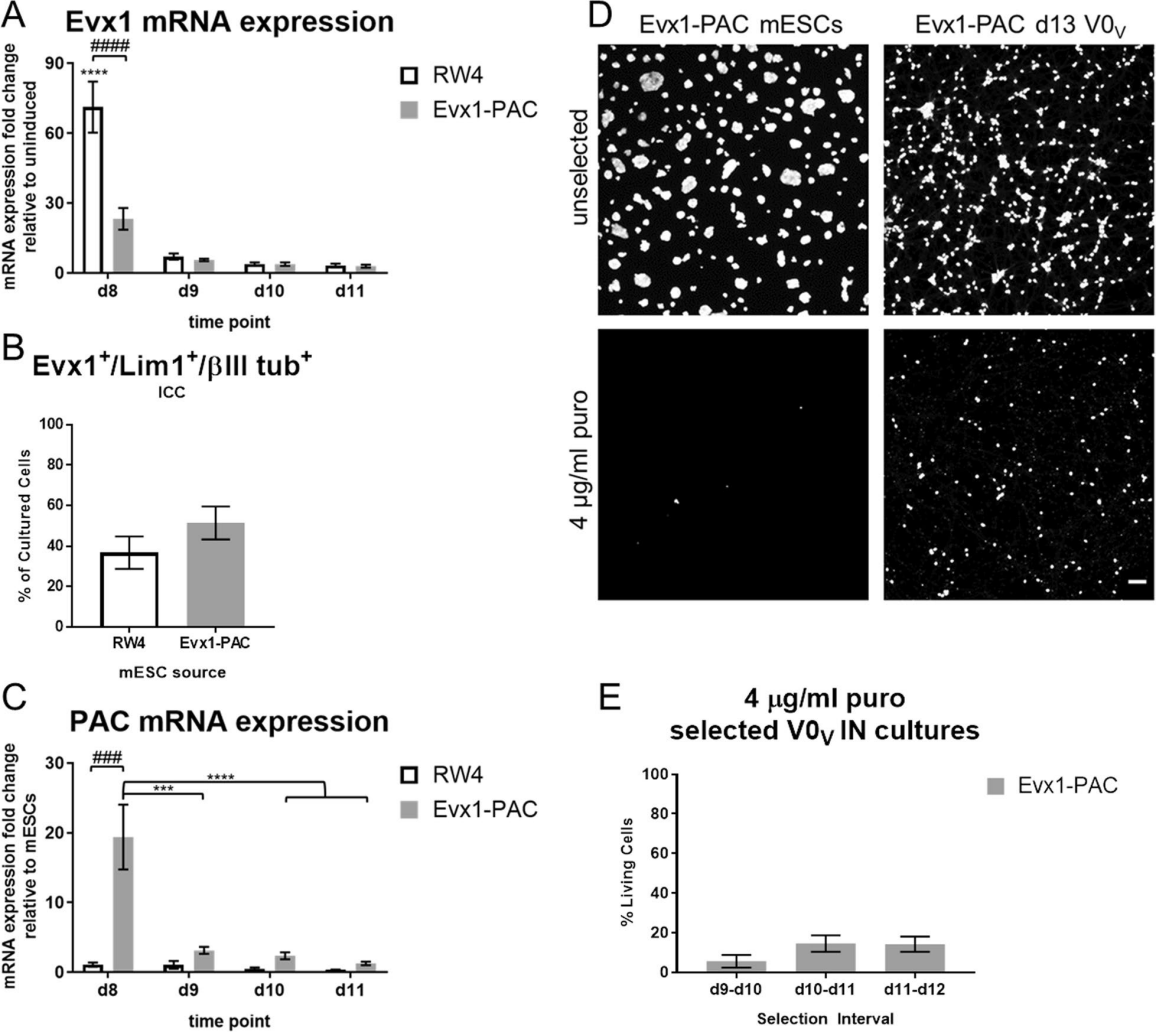
Determining puromycin selection window based on *PAC* and *Evx1* expression. **A** *Evx1* mRNA expression in day 8, 9, 10, and 11 V0_V_ IN induction cultures derived from RW4 or Evx1-PAC mESCs. *N* = 7–8. **B** ICC image analysis showing the percentage of cells expressing V0_V_ IN-markers in RW4- and Evx1-PAC-derived day 11 V0_V_ IN cultures. *N* = 5. **C** *PAC* mRNA expression in day 8, 9, 10, and 11 V0_V_ IN induction cultures derived from RW4 or Evx1-PAC mESCs. *N* = 5–8. **D** Representative images of unselected cultures and cultures selected with 4 µg/ml puro in Evx1-PAC mESCs and Evx1-PAC-derived V0V INs. The V0V IN induction was selected on day 10-11 and imaged on day 13. Scale bar is 50 µm. **E** Percentage of surviving cells after selection with 4 µg/mL puro from day 9–10, day 10–11, or day 11–12, as determined by LIVE/DEAD image analysis (calculated relative to surviving unselected cultures) 2 days after staining (day 12, day 13, or day 14, respectively). *N* = 6. For **A**–**C, E**, error bars are S.E.M. For **A** and **C**, significance is indicated as follows: *** denotes *p* < 0.001 relative to other time points of the same cell line, **** denotes *p* < 0.0001 relative to other time points of the same cell line, ### denotes *p* < 0.001 relative to the same time point for the other cell line, #### denotes *p* < 0.0001 relative to the same time point for the other cell line

Previously we found mESC-derived V0_V_ IN inductions have the highest fold increase in *Evx1* mRNA expression over uninduced cultures at day 8, and the greatest percentage of cells as Evx1^+^/Lim1^+^/βIII tubulin^+^ neurons occurs at day 10 [15]. As *PAC* is inserted into the *Evx1* locus, *Evx1* gene regulatory elements drive expression of *PAC*, and *Evx1* mRNA expression is possibly altered. Both *Evx1* and *PAC* mRNA expression levels were measured to determine whether insertion of *PAC* resulted in its expression while maintaining expression of *Evx1* (Fig. 2). *Evx1* mRNA was found to be significantly decreased in Evx1-PAC-derived V0_V_ IN cultures relative to RW4-derived V0_V_ IN cultures (Fig. 2A). However, image analysis comparing RW4- and Evx1-PAC-derived V0_V_ IN cultures on day 11 showed similar levels of Evx1^+^/Lim1^+^/βIII tubulin^+^ cells (Fig. 2B), with a trend toward a greater percentage of these cells present in Evx1-PAC-derived inductions (25.12 ± 3.58% vs 38.53 ± 8.16% for RW4 vs Evx1-PAC, respectively; *p* = 0.14) demonstrating that even with a reduction in *Evx1* mRNA expression, production of V0_V_ IN marker-expressing cells did not have a corresponding reduction.

Selection of mESC-derived V0_V_ IN cultures should kill mESCs and other cell types while sparing V0_V_ INs. This requires that *PAC* be expressed at a high enough level to achieve selection. In alignment with the window of peak *Evx1* mRNA and protein expression, *PAC* mRNA was examined on days 8, 9, 10, and 11 in V0_V_ IN induction cultures (Fig. 2C). *PAC* mRNA expression was found to have the highest fold increase, around 20-fold relative to mESCs, at day 8. To ensure that the achieved *PAC* mRNA expression level was sufficient for selection, Evx1-PAC mESC cultures and V0_V_ IN cultures were examined for sensitivity to puro (Fig. 2D). The mESCs died in the presence of 4 µg/mL puro. In selected V0_V_ IN cultures, some living cells remained at a lower cell density relative to unselected cultures, showing cell death of some of the heterogenous cultured cells.

After verifying that puro killed mESCs and spared some of the induced cells, the time period for selection was established. 4 µg/mL puro was added to cultures for 24 h on days 9, 10, or 11. After a further 2 days of culture, selected cultures were compared to unselected control cultures plated at the same density at the same time by LIVE/DEAD staining and imaging. A CellProfiler image analysis pipeline was used to determine counts of living cells—only expressing LIVE stain—and the percentage of cells surviving selection was determined by using the count of living cells in selected cultures divided by the count of living cells in unselected cultures (Fig. 2E). Selection on day 10 to 11 or on day 11 to 12 yielded approximately the same survival percentage, around 15%. Based on this survival percentage and the coinciding peak number of V0_V_ IN marker-expressing cells occurring at day 10, cultures were exposed to 4 µg/mL puro on day 10–11 as “selected” cultures for subsequent experiments.

### Examining purity of selected V0_V_ IN cultures

**Fig. 3:**
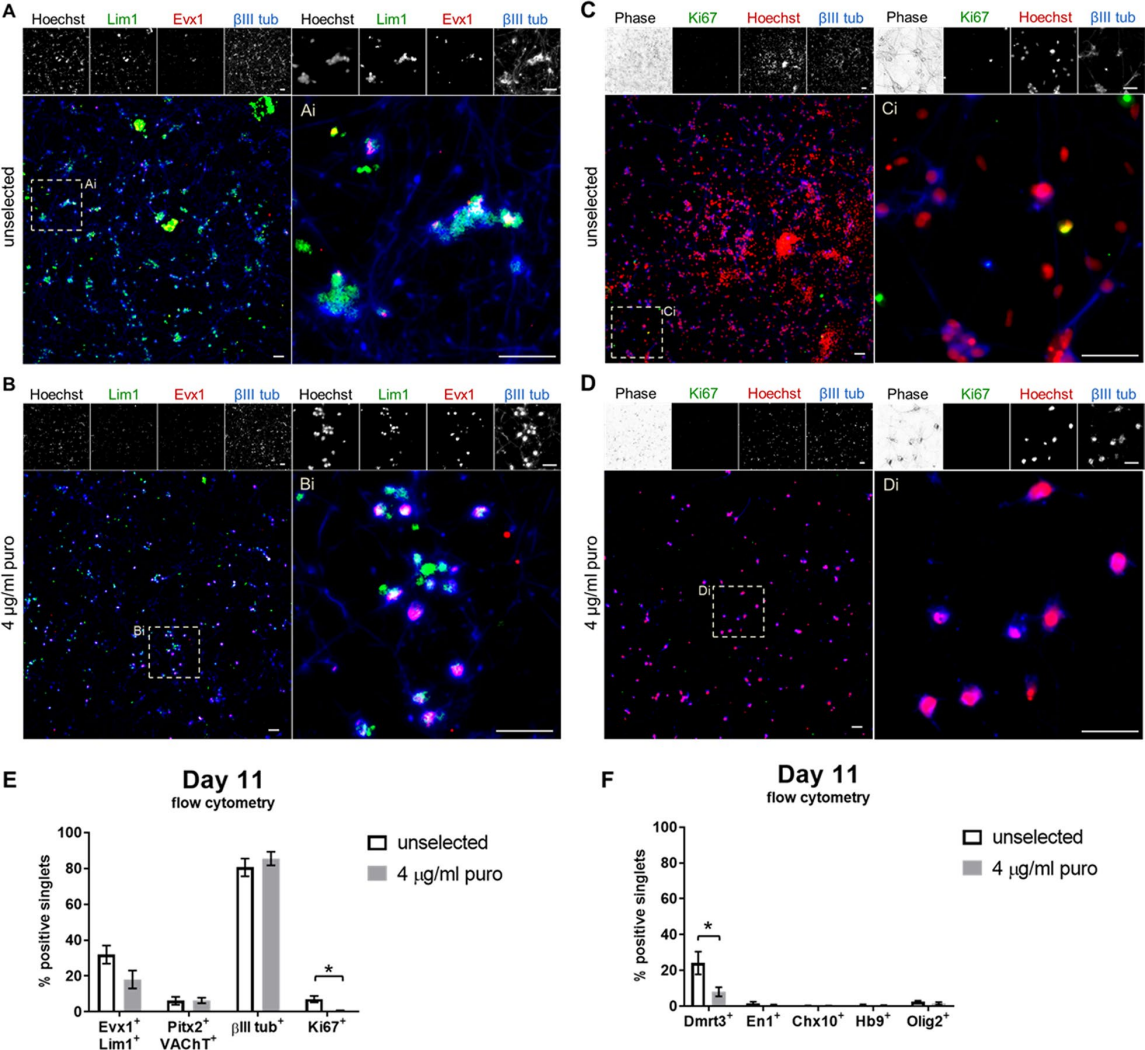
Selected cultures contain V0_V_ IN marker-expressing cells with reduced Ki67^+^ and other IN marker-expressing cells. **A**, **B** Representative images of day 11 Evx1-PAC mESC-derived V0_V_ IN cultures stained by ICC for V0_V_ IN markers Lim1 (green), Evx1 (red), and βIII tubulin (blue). Hoechst stain for nuclei is shown in montage images. **C**, **D** Representative images of day 16 Evx1-PAC mESC-derived V0_V_ IN cultures stained by ICC for proliferative marker Ki67 (green), Hoechst for nuclei (red), and neuronal marker βIII tubulin (blue). Phase is shown in montage images. **A** and **C** show unselected cultures, while **B** and **D** show selected cultures. Scale bars are 50 µm. For each condition, magnified images (Ai, Bi, Ci, Di) are shown adjacent to their derived images to facilitate viewing details. **E**, **F** Day 11 flow cytometry analysis of selected vs unselected Evx1-PAC mESC-derived V0_V_ IN cultures. Error bars are S.E.M., and significance is indicated as follows: * denotes *p* < 0.05 between selected vs unselected cultures for the same stain. **E** Unselected and selected samples were stained for V0_V_ IN markers of Evx1 and Lim1 (N = 13, 15), V0_C_ IN markers of Pitx2 and VAChT (*N* = 4), neuronal marker βIII tubulin (*N* = 11, 13), and proliferative marker Ki67 (*N* = 15, 10). **F** Unselected and selected samples were stained for markers for IN populations arising adjacent to V0_V_ INs: Dmrt3 for dI6 INs (*N* = 11, 10), En1 for V1 INs (*N* = 7, 10), Chx10 for V2a INs (*N* = 10), Hb9 for MNs (*N* = 9, 12), and Olig2 for pMNs and oligodendroglial precursors (*N* = 7)

Selection should ideally spare only V0_V_ INs in the induction cultures, but selection of transgenic cell line-derived neurons by puro does not completely purify the desired population [18, 19]. Evx1 is transiently expressed in vivo and in vitro, and in mESC-derived cultures, the number of cells co-expressing Evx1 and Lim1 that are also identified as neurons from positive βIII tubulin staining decreases dramatically by day 12 [15]. Therefore, although some cell death still occurs over the next couple of days (data observed but not shown; readers are referred to [23]), V0_V_ IN induction cultures were examined by ICC immediately after selection to look at V0_V_ IN markers on day 11 (Fig. 3A, B) to capture the presence of Evx1^+^ cells. During selection, neuronal cells were observed as having fewer and/or shorter neurites, which is not unexpected, as many of the surrounding cells in contact with the surviving cells are dying from exposure to puro. This morphological effect could persist for a day or two before cultures recovered post-selection, and connections remained sparse among the resultant less densely populated surviving cells.

At day 11, there were still some Evx1^+^/Lim1^+^/βIII tubulin^+^ co-expressing cells in both unselected and selected cultures as measured by ICC. Selected cultures appeared to have cells expressing higher levels of Evx1 protein based on observed intensity of staining. Additional file 1: Figure S1 shows quantification for day 11 unselected and selected cultures stained with Evx1, Lim1, and βIII tubulin, suggesting that selection may increase the proportion of Evx1^+^ cells in these cultures. However, the percentage of V0_V_ IN-marker expressing cells was also measured by flow cytometry for unselected and selected cultures immediately after selection on day 11 (Fig. 3E), and there was a trend toward a decrease in the percentage of Evx1^+^/Lim1^+^ cells in selected versus unselected cultures (18.04 ± 5.04% vs. 32.04 ± 5.04% for selected vs unselected, respectively; *p* = 0.062).

These heterogenous V0_V_ IN induction cultures were previously found to have large proportions of non-V0_V_ INs belonging to the populations arising adjacent to V0_V_ INs during development [15]. Markers for these populations were measured by flow cytometry to examine whether they were reduced or eliminated after selection: Dmrt3 for dI6 INs, En1 for V1 INs, Chx10 for V2a INs, Hb9 for MNs, and Olig2 for pMNs and oligodendroglial precursors were used (Fig. 3F). Most of these markers were expressed in very few cells, possibly due to a high percentage of these cells having already become post-mitotic. As such, they may have already downregulated their transiently-expressed definitive transcription factor markers, as induction protocols for other mESC-derived propriospinal neurons yield their respective transcription factor marker-expressing post-mitotic populations after 6 to 8 days [22, 26–28]. For the most adjacent populations of dI6 and V1 INs, Dmrt3^+^ cells significantly decreased from 24.16 ± 6.33% to 8.02 ± 2.57% (*p* = 0.035), while En1^+^ cells decreased from 1.65 ± 0.78% to 0.61 ± 0.25% (*p* = 0.161). V0_V_ INs include a cholinergic Pitx2^+^ subpopulation, V0_C_ INs, which form monosynaptic connections with MNs. Therefore, co-expression of Pitx2 and vesicular acetylcholine transporter (VAChT) was also measured by flow cytometry in selected cultures to compare against unselected cultures (Fig. 3E). There was no difference in the percentage of cells expressing these markers (6.19 ± 2.20% in unselected vs. 6.36 ± 1.54% in selected, *p* = 0.952), even with the measured decrease in cells expressing V0_V_ IN markers.

Proliferative cell types can dilute the post-mitotic neuronal population and result in reduced purity over time and possibly result in teratoma formation after transplantation. Ki67 was used to mark proliferative cells. ICC on day 16 showed few Ki67^+^ cells in unselected cultures (Fig. 3C), while little to no stained cells were shown after selection (Fig. 3D). βIII tubulin was co-stained with Ki67, which showed that neuronal cells did not co-express Ki67, and after selection, neurons persisted, while proliferative cells were removed. Flow cytometry data of day 11 cultures showed that Ki67^+^ cells significantly decreased from 7.06 ± 1.80% to 0.61 ± 0.22% (Fig. 3E; *p* = 0.019). βIII tubulin^+^ cells showed similar or slight increases (80.59 ± 4.94% vs. 85.54 ± 3.80%, *p* = 0.337). Interestingly, the percentage of neuronal cells increased in Evx1-PAC-derived inductions compared to RW4-derived cultures, which had 61.72 ± 7.89% βIII tubulin^+^ cells (data not shown; *N* = 3, *p* = 0.095 vs unselected Evx1-PAC mESC-derived βIII tubulin^+^ cells), perhaps due to altered neuronal specification related to reduced Evx1 expression.

### Observing maturation of long-term selected V0_V_ IN cultures

**Fig. 4:**
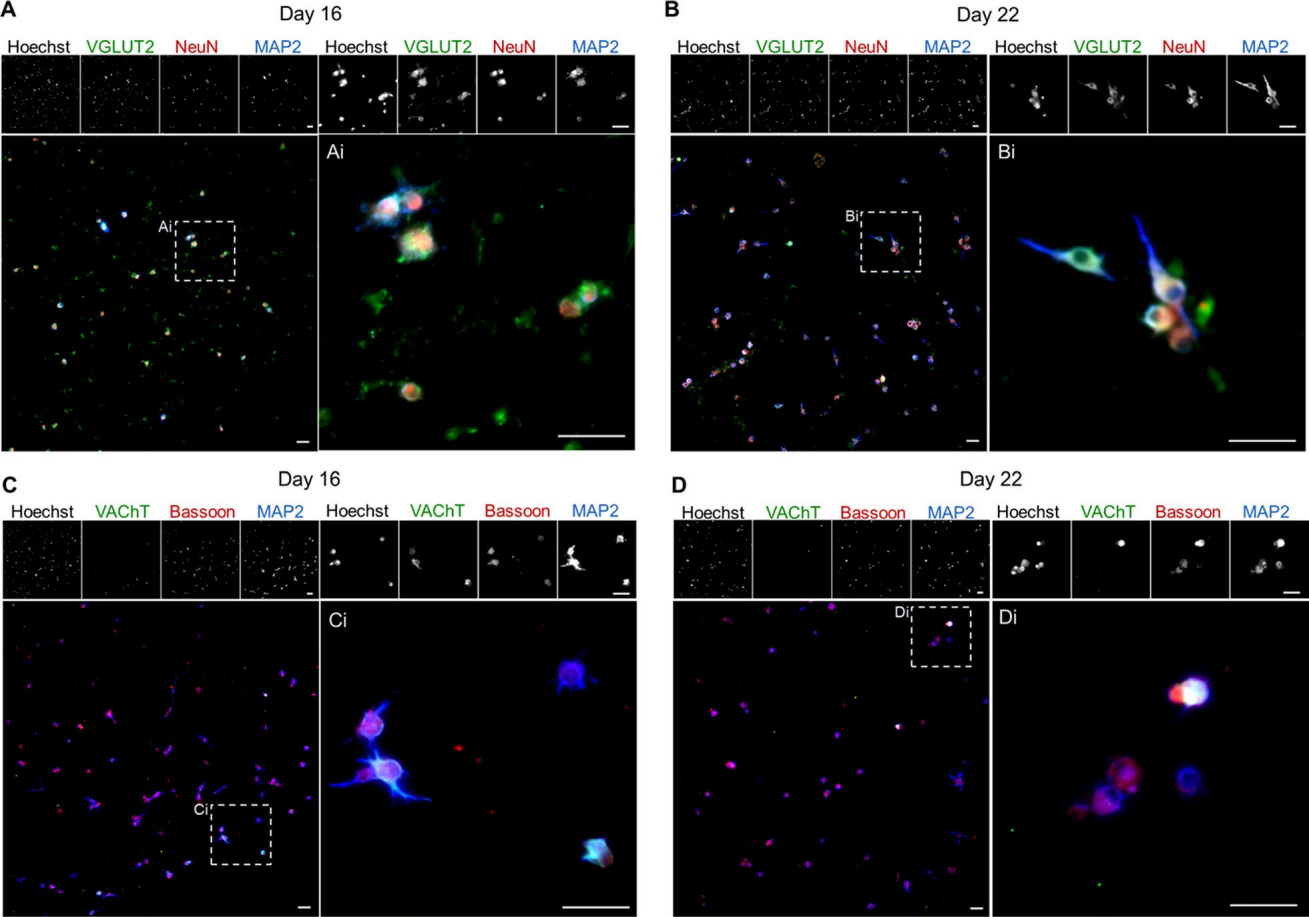
Long-term selected Evx1-PAC mESC-derived V0_V_ IN cultures express mature neuronal markers and synaptic markers. Representative images of selected cultures stained by ICC. **A**, **B** Glutamatergic cell type marker VGLUT2 (green), post-mitotic neuronal marker NeuN (red), and dendritic marker MAP2 (blue). **C**, **D** Cholinergic cell type marker VAChT (green), pre-synaptic marker Bassoon (red), and dendritic marker MAP2 (blue). Hoechst is shown in montage images. Scale bars are 50 µm. For each condition, magnified images (Ai, Bi, Ci, Di) are shown adjacent to their derived images to facilitate viewing details. For **A** and **C**, cultures were examined at day 16, and for **B** and **D**, cultures were examined at day 22

To determine whether selected V0_V_ IN cultures showed maturation over time, cells were examined by ICC for mature neuronal markers and synaptic markers on days 16 and 22 (Fig. 4). Fox3^+^ post-mitotic neuronal nuclei marked by NeuN, microtubule-associated protein 2 (MAP2) as a dendritic marker of maturing neurons, and vesicular glutamate transporter 2 (VGLUT2) for glutamatergic neurons were used to stain cultures (Fig. 4A, B). All three markers were found to be co-expressed in a large proportion of the cells that had survived selection and long-term culture, with clearer processes marked by MAP2 by day 22. Additional file 1: Figure S2A shows quantification of the proportion of cells expressing VGLUT2, NeuN, and MAP2 in day 22 selected cultures. Image analysis shows around 40% of the cells co-expressing VGLUT2 with neuronal markers MAP2 or NeuN.

Potential V0_C_ INs were detected in selected day 16 and day 22 cultures by expression of VAChT; these cells also showed co-expression with the pre-synaptic marker Bassoon and dendritic marker MAP2 (Fig. 4C, D). As flow cytometry data suggest that there are very few or no remaining MNs in selected V0_V_ IN cultures, while Pitx2^+^/VAChT^+^ cells remained, the observed incidence of VAChT^+^ cells additionally supports that there may be V0_C_ INs present. Based on image analysis of ICC data, as seen in Additional file 1: Fig. S2B, around 2% of the selected cells co-express MAP2 and VAChT.

### Measuring selected V0_V_ IN culture activity

Combinations of single population, double population, or triple population co-cultures of mESC-derived selected cultures of V0_V_ INs, MNs, and V2a INs were examined for electrophysiological activity by MEA recordings. Two trials were conducted out to day 30, with one plate having obvious glial presence (+glia), as medium color changed quickly at the later days of culture. Glia were not intentionally included in these selected cultures, but as with any experiment, variability occurs and may have led to selection conditions being more permissive to glial survival. Glial presence was determined by medium color change by rapid acidification and visual assessment of cell morphology, which did not occur in cultures with only neurons present. Acidification did not allow for culture beyond 30 days without requiring more frequent media changes to avoid over-acidification of the cultures. Although not intended, cultures with glia were maintained because of the observed differences in firing activity.

**Fig. 5:**
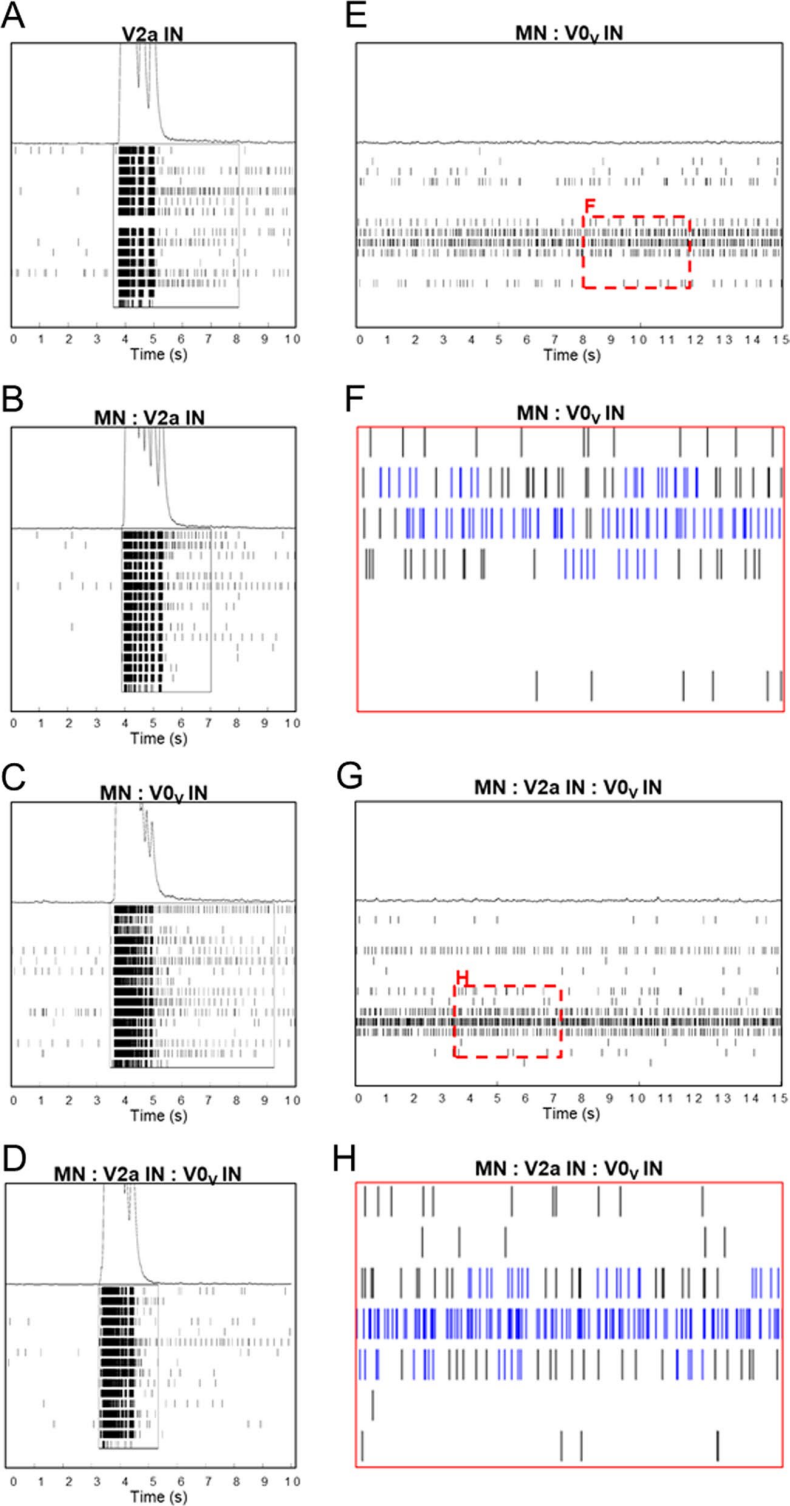
Bursting patterns of networks formed from combined, selected V0_V_ INs, V2a INs, and MNs. **A**–**D** Bursting patterns of selected IN and MN cultures on day 26 of Evx1-PAC mESC-derived V0_V_ IN cultures which were surmised to contain glia: **A** V2a IN, **B** MN:V2a IN co-cultures, **C** MN:V0_V_ IN co-cultures, **D** MN:V2a IN:V0_V_ IN co-cultures. A 10 s time period is shown, and boxes within plots represent Axion’s software indication of a network burst. **E**–**H** Bursting patterns of co-cultures containing day 26 Evx1-PAC mESC-derived V0_V_ INs with little glial presence: **E**,** F** MN:V0_V_ IN co-culture, **G**, **H** MN:V2a IN:V0_V_ IN co-culture. **F** and **H** are snippets from **E** and **G**, respectively. For **E** and **G**, a 15 s time period is shown. In **F** and **H**, blue bars show Axion’s software indication of bursts versus black bars which are spikes without bursting. Some semblance of alternation can be detected in both co-culture conditions

Some but not all populations showed bursting activity. Bursting patterns at day 26 for those populations that had bursting activity are shown in Fig. 5. Bursting patterns of the very robust bursts that occurred in day 26 cultures +glia are shown in Fig. 5A–D. V0_V_ IN cultures did not show bursting when cultured alone but had bursts in co-cultures with MNs or MNs and V2a INs V2a INs show distinct, synchronized short bursts within the network burst (Fig. 5A); this pattern is also observed in the MN:V2a IN co-cultures (Fig. 5B). In MN:V0_V_ IN cultures, there is a longer train of bursting before any distinct, shorter bursts are observed (Fig. 5C). When MN:V2a IN:V0_V_ INs are cultured together, this burst train still occurs but is shorter and is followed by the distinct short bursts seen in V2a IN cultures (Fig. 5D). These distinct patterns support the idea that different excitatory populations—V2a and V0_V_ INs—are present and communicating differently with MNs, and when all are present, both IN populations contribute to the network circuitry. This sort of synchronized bursting was observed less frequently, if at all, in the MEA cultures with little glial presence (not shown). Although cultures without glia had less synchronized bursting, patterns reminiscent of alternation occurred in cultures that contained V0_V_ INs (Fig. 5E–H). These “alternations” were observed from around day 24 to around day 28.

**Fig. 6:**
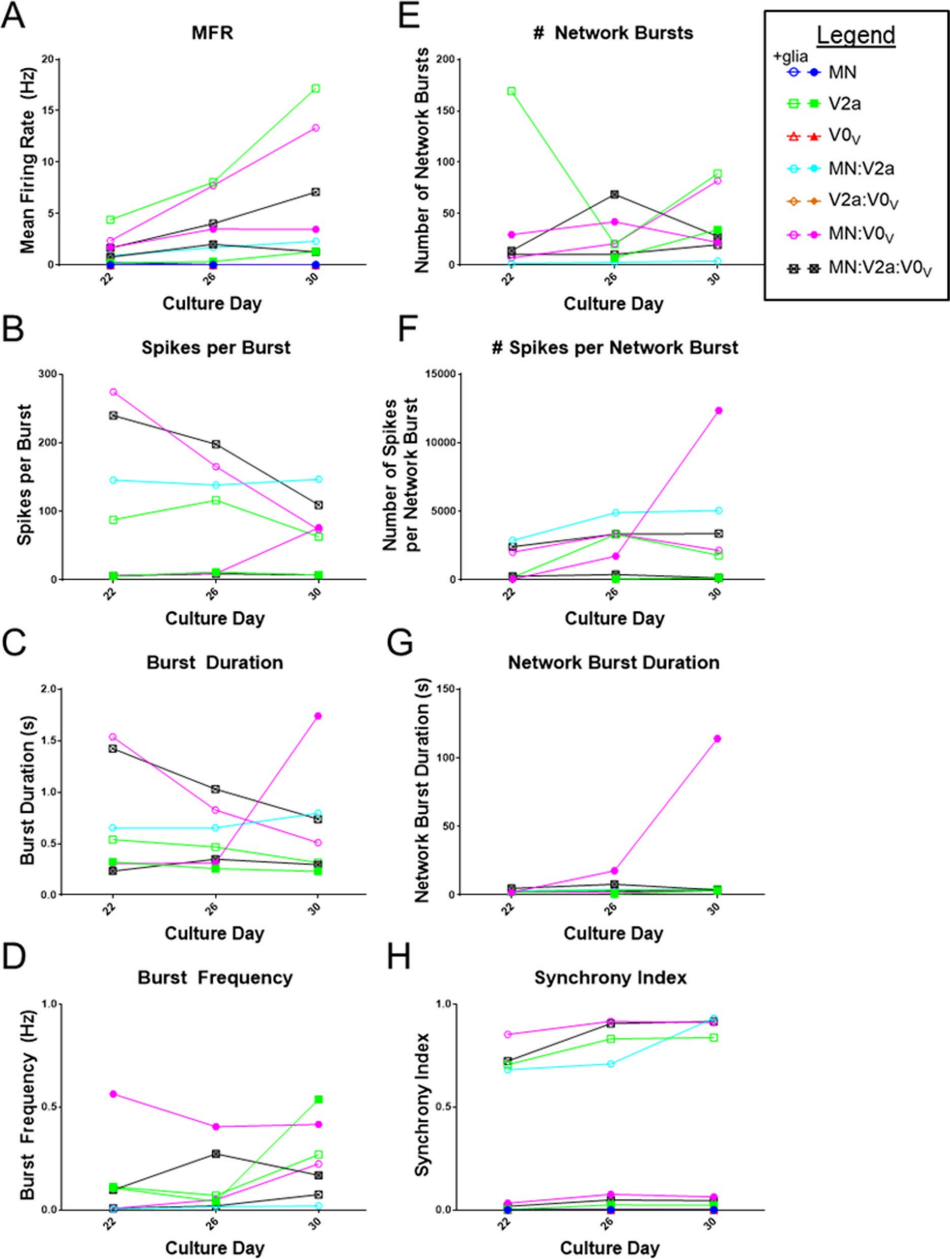
Activity and network metrics of selected co-cultures of V0_V_ INs, V2a INs, and MNs. **A**–**D** Metrics of different populations at day 22, 26, or 30 of V0_V_ IN culture. Activity metrics include **A** mean firing rate (MFR) in Hz, **B** spikes per burst, **C** burst duration in seconds, and **D** burst frequency in Hz. Network metrics include **E** number of network bursts, **F** number of spikes per network burst, and **G** network burst duration in seconds. Synchrony metrics include **H** the synchrony index, a unitless measurement indicating perfect synchrony at 1 and perfect asynchrony at 0. A legend shows symbols for each population alone, double populations, and all three cell types together, both in the culture with (open symbols, +glia) and without (closed symbols) glia: MN—blue circles, V2a IN—green squares, V0_V_ IN—red triangles, MN:V2a IN—cyan hexagons, V2a IN:V0_V_ IN—orange diamonds, MN:V0_V_ IN—magenta hexagons, MN:V2a IN:V0_V_ IN—gray/crossed squares

Figure 6 shows activity, network, and synchrony metrics from day 22, 26, and 30 MEA recordings. Activity and synchrony metrics are shown for all population combinations, while bursting metrics are only shown for cultures that displayed bursting, which did not include V0_V_ INs, MNs, or V2a:V0_V_ IN co-cultures. To highlight the differences in firing for cultures with no obvious glia versus those with surmised glial presence, data are presented for each replicate rather than as means. The mean firing rate (MFR) of either MNs or V0_V_ INs alone or V2a IN:V0_V_ IN co-cultures did not increase above what occurred at the initial plating (on day 12) over time (Fig. 6A), which was sporadic and therefore could be considered background just above the set threshold for spike detection. Otherwise, the general trend was that MFR increased over time, and MFR for cultures in the presence of glia were higher than without glia. MN:V0_V_ IN cultures had a greater MFR than the MN:V2a IN or MN:V2a IN:V0_V_ IN co-cultures. MN:V2a IN cultures only had a MFR above background when glia were present.

Only cultures that had any increase in MFR over background levels observed immediately after plating also had any bursting (V2a IN, MN:V2a IN, MN:V0_V_ IN, MN:V2a IN:V0_V_ IN). Co-cultures containing V0_V_ INs +glia had a larger number of spikes per burst which decreased over time (Fig. 6B). Spikes per burst for V2a IN cultures +glia and co-cultures with MNs +glia remained level over time. Burst durations for cultures +glia also decreased or remained level (Fig. 6C), coinciding with the reduced number of spikes per burst over time. These data also align with the burst frequency remaining level over time (Fig. 6D). Of note are that by day 30, the V2a IN culture burst frequency increases, while the MN:V0_V_ IN co-cultures have an increased burst duration and spikes per burst; these data may allude to the IN populations’ roles in the network.

## Discussion

CRISPR/Cas9 technology was used to generate a DSB for HDR-mediated insertion of PAC into a single *Evx1* locus. Two *Evx1* gRNAs, guide 21 and guide 28, were designed and delivered to electroporated mESCs, but the clones derived from guide 28 resulted in much higher levels of *PAC* mRNA expression in the mESCs (data not shown), which made it difficult to detect a fold change increase in *PAC* mRNA expression in V0_V_ IN inductions relative to mESCs. Likely, this would have resulted in poor selection of V0_V_ INs from cultures and/or remaining mESCs post-selection. Therefore, clones chosen for further screening were all from electroporation with guide 21, which produced little *PAC* mRNA in mESCs.

After insertion of *PAC* into *Evx1*, there was a significant decrease in the amount of *Evx1* mRNA expression in V0_V_ IN inductions derived from the Evx1-PAC mESC line compared to RW4-derived inductions. However, there was a considerable, although not significant, increase in the proportion of induced cells co-expressing the V0_V_ IN markers Evx1, Lim1, and pan-neuronal marker βIII tubulin (Fig. 2B). There was also an increase in the percent of βIII tubulin^+^ cells in Evx1-PAC mESC-derived cultures compared to RW4 mESC-derived cultures, as measured by flow cytometry (data not shown). This suggests that disruption of one *Evx1* allele results in altered neuronal specification, but the mechanism behind this change is unclear and requires further study. Previous work using mutated *Evx1* showed it has involvement in anteroposterior patterning through associated BMP/Wnt pathway signaling effects [29]. Evx1 mutation resulted in downregulated expression of *BMP* and *Wnt* pathway genes; as the proteins of these genes are known to be involved in dorsal IN specification [30], perhaps Evx1-PAC mESCs can more easily generate ventral IN subtypes, resulting in the observed increase of V0_V_ IN marker-expressing cells.

The puro selection window was determined to be 24 h from day 10 to 11 or day 11 to 12. All analyses for selected culture composition, maturation, and functional recordings were completed with day 10 to 11 selected cultures. It is possible the results of these analyses would differ if day 11 to 12 cultures were examined instead, such as by allowing more time for V0_C_ IN specification before selecting, for example. Future work is needed to determine if the alternate selection window changes the measured outcomes for these cultures.

Evx1-expressing cells appeared to be enriched in selected versus unselected cultures as observed by ICC (Fig. 3A, B, and Fig. S1). However, flow cytometry data showed that there was an obvious, although not significant, decrease in Evx1^+^/Lim1^+^ cells in selected cultures. It is possible that the robust Evx1 expression observed by ICC is due to those Evx1^+^ surviving cells expressing relatively higher levels of Evx1 compared to others that succumbed to puro, and that the discrepancy between ICC and flow data is due in part to this relatively higher expression as well as the transient expression of Evx1 known to occur in these cells [11].

To further illuminate, consider the following potential explanation. On day 11, Evx1 is expressed in about 30% of the induced cells, some with higher expression levels than others (this percentage would be slightly higher at day 10 and lower at day 12 due to transient Evx1 expression). This is likely due to the use of EB-based inductions resulting in “waves” of V0_V_ IN specification, the foremost wave starting around day 8, followed by successive waves that build up to yield the peak of cells expressing V0_V_ IN markers at day 10, and continue until about day 12 when marker-expressing cells wane. Of the 30% Evx1^+^ day 11 cells, those from earlier waves might already be turning Evx1 expression off, thus having a concomitant decrease in PAC expression. These cells expressing lower levels succumb to puro, while those with higher Evx1/PAC levels from waves closer to the time of selection survive. Some of the V0_V_ INs that survived selection might, at the time of assaying, have low Evx1 expression or have already turned off Evx1 expression, while the ones retaining Evx1 expression have high levels of expression. Therefore, flow cytometry measurements might not be a true representation of the surviving percentage of V0_V_ INs these cultures. Further work is needed to verify that the surviving cells are truly V0_V_ INs, perhaps by finding targets downstream of Evx1 and examining their expression in these cells or by generating an alternative, lineage-traceable mESC line.

Although verification of selection yielding purification of cells expressing definitive V0_V_ IN markers was not achieved, ICC staining showed that surviving cells were largely glutamatergic, as are V0_V_ INs, and included some VAChT^+^ cells as putative V0_C_ INs. Previous work shows the largest proportion of non-V0_V_ IN marker-expressing cells in these cultures are Dmrt3^+^ or En1^+^ neurons [15]. These data are indicative that surviving neurons are V0_V_ INs, as dI6 INs and V1 INs are both inhibitory populations [31, 32].

In MEA functional studies, of the single population cultures, V2a INs were the only population that achieved bursting. This could possibly be due to survival of other propriospinal cell types, such as MNs, V2b INs, or even V0_V_ INs, which arise close to V2a INs and have been shown to be part of the heterogenous resultant mESC-derived V2a IN culture, even after selection with 2 µg/mL puro, albeit at very low percentages [18, 22]. However, V2a INs purposely co-cultured with either V0_V_ INs or MNs did not achieve bursting, except for with MNs when glia were present. Co-culture with MNs +glia also did not achieve as high a level of activity as V2a INs cultured alone, suggesting that inhibitory populations such as V2b INs are not likely to be present, and possibly, if there are MNs in the V2a IN only cultures, the proportion of MNs must remain low relative to V2a INs to achieve higher bursting activity. Also, previous work in our laboratory showed that these mESC-derived selected V2a IN cultures could synapse with each other [18]. Studies examining the electrophysiological characteristics of V2a INs in vivo described three electrophysiological classes of V2a INs, and those of the same class can undergo electrical coupling and fire rhythmically during fictive locomotion [33]. This also aligns with the observed bursting pattern of V2a IN single population cultures having distinct, rhythmic short bursts at the late stage of the network burst (Fig. 5A), as well as contributing a rhythmic bursting pattern to MN co-cultures (Fig. 5B).

Examining recordings of the different combinations of selected V2a INs, MNs, and V0_V_ INs allowed some light to be shed on their role in the generated networks. Even with variable data within and among groups, a general idea of the activity of the different populations can be gleaned. The presence of glia appears to support network formation, as cultures with glia showed improved maturation based on MFR, burst metrics, and greater network synchrony; these results could be anticipated based on previous work showing glial support of network formation and maturation [34]. The difference in bursting for cultures containing V0_V_ INs with (Fig. 5C, D) versus without glia (Fig. 5E–H) could suggest that some population of V0_V_ IN contributing to alternation is not as reliant on glial support for maturation, while another population that contributes to burst trains observed in cultures +glia requires glial support for survival and/or maturation. It is exciting to think these could be V0_C_ INs, which are involved in MN output modulation [14], as the burst trains suggest some level of burst modulation through the extended burst duration. ICC shows VAChT^+^ cells are present; however, further work is needed to verify these are the cells that contribute to this bursting pattern in these cultures. More replicates, both with and without glia, might help further elucidate MN and IN functions in MEA networks, but a recent analysis of MEA-based data suggests that many—perhaps even an impractical number, depending on the complexity of the culture and activity level achieved—replicates would be needed to obtain statistically relevant data due to batch-to-batch variability and other factors influencing MEA measurements [35].

In studies examining CPG circuitry using spinal cord preparations, the native circuitry is organized, resulting in clear pairs of alternators, whether flexor–extensor or left–right pairs. However, in these in vitro cultures, there are not necessarily distinct pairs but, as appears to be the case observed in Fig. 5F, H, there could be three or more alternators communicating with each other. This may seem counterproductive if these cells were to be transplanted into injured spinal cords with the aim of improving motor function, since motor functions require pairing and additional nodes may be introduced by these cells. However, it might be possible that the remaining organized tissue after injury would incorporate and lend organization to any transplanted cells, especially since supportive glia and other circuitry components like inhibitory INs may contribute to and guide their activity post-transplantation.

In this paper we have shown the successful creation of a Evx1-PAC transgenic mESC line with a single modified locus to use as a tool in selected IN circuit studies and for transplantation studies in rodent models. V0_V_ IN markers were detected, although not at enriched levels after selection—likely due to transient Evx1 expression. The surviving INs were largely neurons expressing glutamatergic marker VGLUT2 and showing maturation and synaptic markers in long-term cultures. A proportion of maturing cells also showed expression of VAChT, a marker for the V0_V_ subpopulation, cholinergic V0_C_ INs, which normally modulate MN output. In terms of functionality, the selected Evx1-PAC mESC-derived V0_V_ cultures formed connections with MNs or V2a INs and MNs that resulted in synchronized network bursting in the presence of glia and ostensible alternating activity in V0_V_ IN co-cultures without glia. This transgenic mESC line is a valuable tool for controlled experiments exploring locomotor CPG circuitry and potentially as a therapeutic option in animal studies.

## Conclusions

This work has shown the successful generation of a transgenic Evx1-PAC mESC line to allow for purification of a glutamatergic neuronal population. After selection with puromycin from day 10–11, cultures show removal of other IN populations and proliferative cells, thus enriching cultures for V0_V_ INs. Long-term selected cultures express mature neuronal markers, and when co-cultured with MNs and V2a INs, these selected cultures show functional network activity. These data recommend this cell line as a useful tool for in vitro network studies and in vivo rodent models investigating recovery.

## Supplementary Information

**Additional file 1: Fig. S1.** Day 11 quantification of Evx1, Lim1, and βIII tubulin by ICC image analysis. V0_V_ IN induction cultures were either unselected or selected with 4 µg/ml puro from day 10 to 11, then fixed and stained on day 11. N = 1 with n = 2 for unselected and n = 5 for selected cultures. Error bars are S.E.M. among technical replicates. Figure S1 is associated with Fig. 3. **Additional file 1: Fig. S2.** Day 22 quantification of mature neuron and synaptic markers by ICC image analysis. V0_V_ IN induction cultures were selected with 4 µg/ml puro from day 10 to 11 and cultured until day 22, when they were fixed and stained. Percentage of selected cultured cells expressing **A** VGLUT2, NeuN, and MAP2 or **B** VAChT, Bassoon, and MAP2 and respective co-staining combinations are shown. N = 2 with n = 3–5 and error bars are S.E.M. Figure S2 is associated with Fig. 4.

## Data Availability

The datasets used and/or analyzed in this study are available from the corresponding author upon reasonable request.

## Abbreviations

INs: Interneurons
CNS: Central nervous system
V0_V_: Ventral V0
ESCs: Embryonic stem cells
mESCs: Mouse embryonic stem cells
PAC: Puromycin-*N*-acetyltransferase
MNs: Motor neurons
MEAs: Microelectrode arrays
SCI: Spinal cord injury
V0_D_: Dorsal V0
PSCs: Pluripotent stem cells
iPSCs: Induced pluripotent stem cells
CM: Complete medium
DMEM: Dulbecco’s Modified Eagle Medium
NB: Neurobasal medium
BDNF: Brain-derived neurotrophic factor
BSA: Bovine serum albumin
PBS: Phosphate buffered saline
GDNF: Glial cell line-derived neurotrophic factor
NT-3: Neurotrphin-3
LIF: Leukemia inhibitory factor
BME: β-Mercaptoethanol
EDTA: Ethylenediaminetetraacetic acid
NEB: New England Biolabs
rSAP: Shrimp alkaline phosphatase
jPCR: Junction polymerase chain reaction
EB: Embryoid body
RA: Retinoic acid
purm: Purmorphamine
DMSO: Dimethyl sulfoxide
DAPT: *N*-{*N*-(3,5-Difluorophenacetyl-l-alanyl)}-(*S*)-phenylglycine-t-butyl-ester
puro: Puromycin
HBSS: HEPES-buffered saline solution
PFA: Paraformaldehyde
NGS: Normal goat serum
ICC: Immunocytochemistry
S.E.M.: Standard error of the mean
ANOVA: Analysis of variance
HDR: Homology-directed repair
gRNA: Guide RNA
DSB: Double-stranded break
TK3: Thymidine kinase 3
n.e.: Neo-excised
VAChT: Vesicular acetylcholine transporter
MAP2: Microtubule-associated protein 2
VGLUT2: Vesicular glutamate transporter 2
MFR: Mean firing rate
