# Supplementary material for: A transgenic mouse embryonic stem cell line for puromycin selection of V0V interneurons from heterogenous induced cultures
Source: Stem Cell Res Ther. 2022 Mar 28;13:131. doi: 10.1186/s13287-022-02801-7 (PMC8962475; doi:10.1186/s13287-022-02801-7)
Supplement: Supplementary file 1 — Additional file 1: Fig. S1. Day 11 quantification of Evx1, Lim1, and βIII tubulin by ICC image analysis. V0V IN induction cultures were either unselected or selected with 4 µg/ml puro from day 10 to 11, then fixed and stained on day 11. N = 1 with n = 2 for unselected and n = 5 for selected cultures. Error bars are S.E.M. among technical replicates. Figure S1 is associated with Fig. 3. Additional file 1: Fig. S2. Day 22 quantification of mature neuron and synaptic markers by ICC image analysis. V0V IN induction cultures were selected with 4 µg/ml puro from day 10 to 11 and cultured until day 22, when they were fixed and stained. Percentage of selected cultured cells expressing A VGLUT2, NeuN, and MAP2 or B VAChT, Bassoon, and MAP2 and respective co-staining combinations are shown. N = 2 with n = 3–5 and error bars are S.E.M. Figure S2 is associated with Fig. 4. [file 13287_2022_2801_MOESM1_ESM.docx]

Figure S1


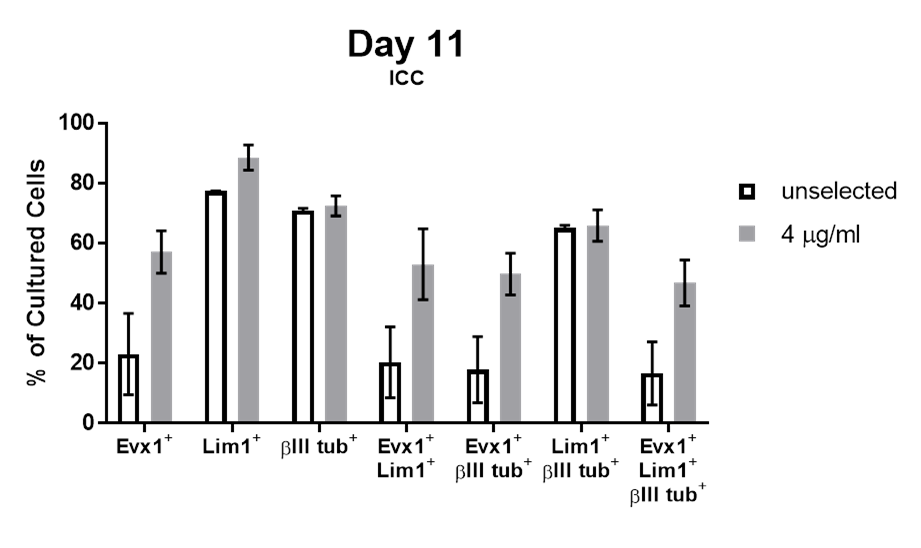


**Figure S1: Day 11 quantification of Evx1, Lim1, and βIII tubulin by ICC image analysis**

V0_V_ IN induction cultures were either unselected or selected with 4 µg/ml puro from day 10 to 11, then fixed and stained on day 11. N = 1 with n = 2 for unselected and n = 5 for selected cultures. Error bars are S.E.M. among technical replicates. Figure S1 is associated with Figure 3.

Figure S2


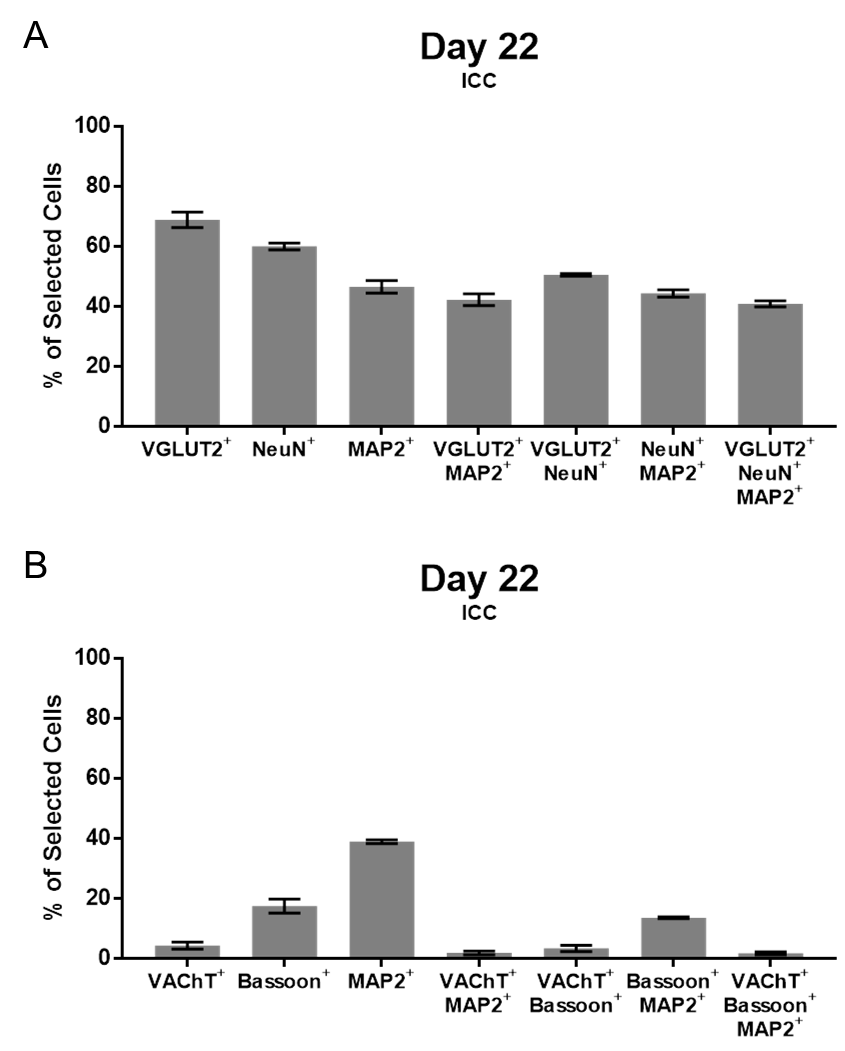


**Figure S2: Day 22 quantification of mature neuron and synaptic markers by ICC image analysis**

V0_V_ IN induction cultures were selected with 4 µg/ml puro from day 10 to 11 and cultured until day 22, when they were fixed and stained. Percentage of selected cultured cells expressing **(A)** VGLUT2, NeuN, and MAP2 or **(B)** VAChT, Bassoon, and MAP2 and respective co-staining combinations are shown. N = 2 with n = 3 – 5 and error bars are S.E.M. Figure S2 is associated with Figure 4.
